# Changes across the psychometric function following perceptual learning of an RSVP reading task

**DOI:** 10.3389/fpsyg.2014.01434

**Published:** 2014-12-23

**Authors:** Daniel R. Coates, Susana T. L. Chung

**Affiliations:** ^1^Vision Science Graduate Program, University of CaliforniaBerkeley, CA, USA; ^2^School of Optometry, University of CaliforniaBerkeley, CA, USA

**Keywords:** perceptual learning, reading, rapid serial visual presentation, central vision loss, psychometric functions

## Abstract

Several recent studies have shown that perceptual learning can result in improvements in reading speed for people with macular disease (e.g., Chung, 2011; Tarita-Nistor et al., 2014). The improvements were reported as an increase in reading speed defined by specific criteria; however, little is known about how other properties of the reading performance or the participants' perceptual responses change as a consequence of learning. In this paper, we performed detailed analyses of data following perceptual learning using an RSVP (rapid serial visual presentation) reading task, looking beyond the change in reading speed defined by the threshold at a given accuracy on a psychometric function relating response accuracy with word exposure duration. Specifically, we explored the statistical characteristics of the response data to address two specific questions: was there a change in the slope of the psychometric function and did the improvements in performance occur consistently across different word exposure durations? Our results show that there is a general steepening of the slope of the psychometric function, leading to non-uniform improvements across stimulus levels.

## 1. Introduction

Performance for a variety of visual tasks improves with practice. This improvement is often termed *perceptual learning* and can be observed in the normal fovea (e.g., McKee and Westheimer, 1978; Ball and Sekuler, 1982, 1987; Karni and Sagi, 1991; Poggio et al., 1992; Fahle and Edelman, 1993; Li et al., 2004; Lu and Dosher, 2004) and the periphery (e.g., Chung et al., 2004, 2005; Chung, 2007). The effectiveness of perceptual learning in improving visual performance in the periphery is particularly important in relation to visual rehabilitation because it is commonly believed that the properties of vision in people with strabismic amblyopia resemble those of the normal periphery (Levi, 1991; Levi and Carkeet, 1993), and that people who lose their central vision due to macular disease must use their peripheral vision for seeing. Indeed, perceptual learning has been used as a remedy to improve functional vision for people with amblyopia for over two decades (for reviews, see Levi, 2005, Levi and Li, 2009 or Astle et al., 2011). Only recently have we observed an intense interest in applying perceptual learning to improve functional vision in people with central vision loss. Enhancing reading performance is a central goal, likely because the majority of patients seeking low vision rehabilitation services had central vision loss and most of them complained of reading difficulties (Owsley et al., 2009).

Previously, we tested the feasibility of using perceptual learning to improve reading speed for a group of six participants with long-standing macular disease: age-related macular degeneration (AMD) or Stargardt disease (Chung, 2011). Our task involved presenting sentences one word at a time using the rapid serial visual presentation (RSVP) paradigm and measuring reading accuracy as a function of word exposure duration. The advantage of using RSVP to train people with macular disease is that RSVP minimizes the need to make intra-word saccades during reading (Rubin and Turano, 1994), thus the training is not contaminated by any potential deficiency in eye movements, as has been reported for these individuals (White and Bedell, 1990). As such, the RSVP paradigm also allows us to independently test whether eye movement training or reading training would be more beneficial to people with macular disease, as in the study of Seiple et al. (2011). For example, it is hypothesized that “crowding” between letters in the periphery limits reading speed, and it has been shown previously that peripheral letter crowding can be reduced with perceptual learning (Chung et al., 2004; Chung, 2007).

Using our method, we defined reading speed based on the word exposure duration that yielded 80% correct on the psychometric function (PF) relating reading accuracy with word exposure duration. Our result showed that reading speed improved by an average of 53% following 6 weekly sessions of training using an RSVP reading task. Nguyen et al. (2011) trained a group of Stargardt disease patients also using an RSVP reading task and reported an increase of 25% of the median reading speed of the group following training. More recently, Tarita-Nistor et al. (2014) reported a 54% improvement in reading speed following training with a print size close to the threshold print size. In all these studies, reading speed was the primary measurement during training, and was used to gauge the effectiveness of the training paradigm. Besides the improvement in reading speed, which often was defined based on the shortest amount of time to read at a given level of accuracy, little is known about whether or not, and how, perceptual learning alters other properties of the participants' reading responses. Did the improvement in reading speed occur only at a specific testing duration, or did it generalize to other durations?

To further probe the effects of perceptual learning on the properties of participants' reading responses, we need to be able to fully characterize participants' reading performance at different stimulus levels (reading durations) and/or accuracy levels. The approach in our previous paper, measuring reading performance using the method of constant stimuli for multiple word durations, and fitting a psychometric function of reading accuracy vs. stimulus duration to describe the data (Chung, 2011), offers us the opportunity to explore the statistical characteristics of the response data beyond the defined reading speed thresholds. Other approaches have also been used to measure RSVP reading performance, most commonly using adaptive methods (Nguyen et al., 2011; Seiple et al., 2011; Tarita-Nistor et al., 2014). These approaches target at trials around a given accuracy criterion (the “threshold”) and do not lend themselves readily for analyses beyond giving us the threshold values. In this paper, we are specifically interested in two questions: (1) was there a change in the slope of the psychometric function as a result of perceptual learning, and (2) were the performance improvements uniform across different word exposure durations?

To address our questions, we performed detailed analyses on the dataset of Chung (2011), with data from an additional new participant, also with age-related macular degeneration (AMD). Because a psychometric function was used to fit the data from each training block, we could evaluate if the slope of the psychometric functions change over the learning process (Question 1). An understanding of changes in the slope of the psychometric function is critical for at least two reasons. First, assumptions about the slope are the theoretical basis of adaptive methods such as QUEST (Watson and Pelli, 1983; Kontsevich and Tyler, 1999). Second, changes in the slope of the underlying psychometric function with learning may provide information about the underlying mechanism of the learning process or the specificity of the learning effect.

**Figure 1:**
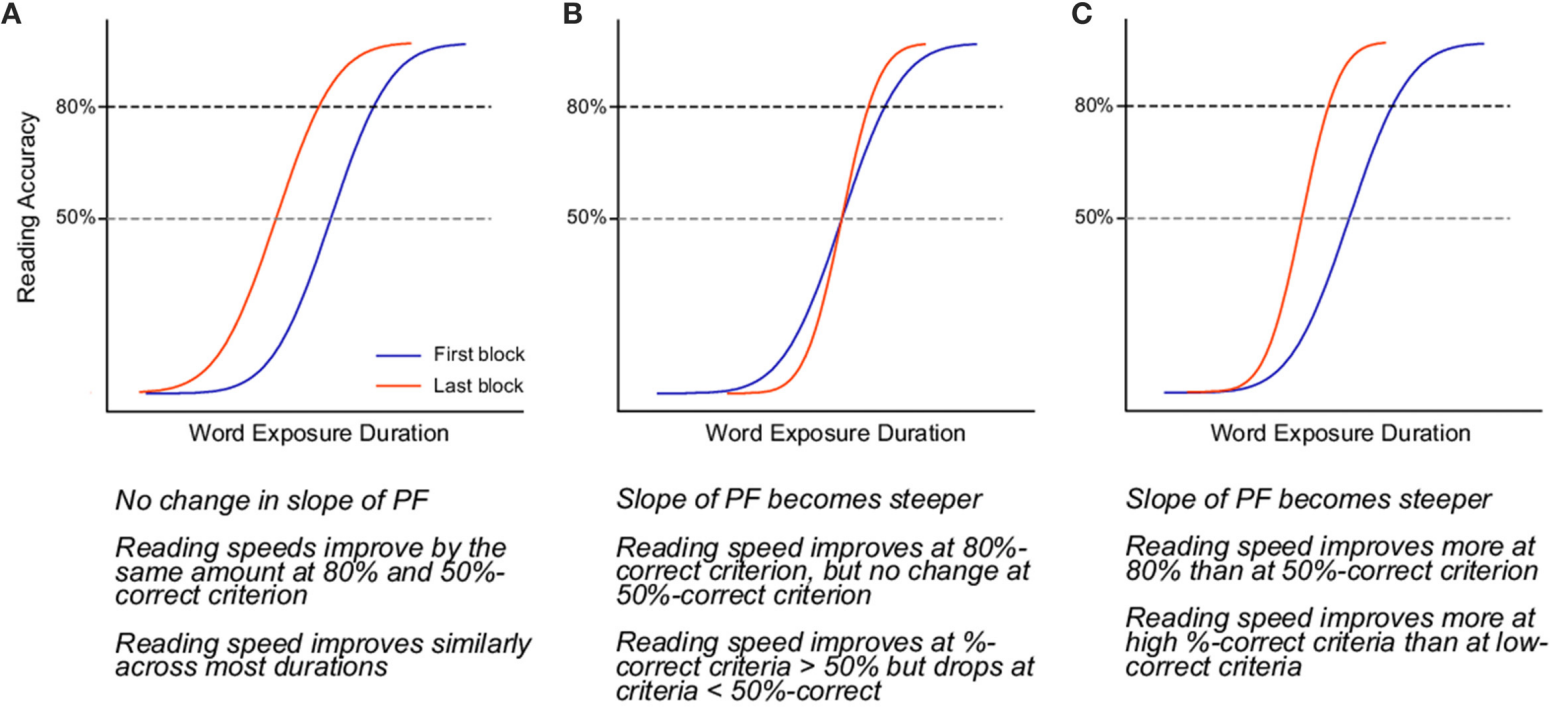
**Panels A–C illustrate the three possible outcomes of perceptual learning**.

The predictions of how a psychometric function relating reading accuracy and stimulus duration may change as a result of perceptual learning are shown in Figure 1. The blue and red curves in each panel represent the psychometric function before and after training, respectively. Here, we make two general assumptions of the effects of perceptual learning on reading performance: (1) reading speed improves, which means that at the same level of accuracy, words can be read at shorter durations after training than before; and (2) the slope of the psychometric function either remains the same or becomes steeper after training, but will not become shallower. The three scenarios in Figure 1 summarize how these effects may combine to produce the observed changes in the psychometric function following training. Panel A shows the scenario in which the slope of the psychometric function (sensitivity of responses) does not change. In this case, improvements in reading performance appear as a mere leftward shift of the psychometric function toward shorter durations (corresponding to faster reading speeds), yielding similar magnitudes of improvements across all durations, except at the very low and high end of the psychometric function. Panel B represents the case in which only the slope of the psychometric function becomes steeper (improvement in response sensitivity) following training. Because the slope of a psychometric function is defined with respect to the mean of the function (the 50% point), a steepening of the psychometric function (without any horizontal shift) would appear as an improvement in reading speed for accuracy levels above the mean of the psychometric function. However, this scenario predicts that there will be a drop in reading speed corresponding to accuracy levels below the 50% point. In Panel C, the steeper psychometric function is also accompanied by a leftward shift, resulting in improvements in reading speed that differ depending on the accuracy levels. For instance, reading speed defined at an 80% correct accuracy would yield a larger improvement than at 50% correct.

## 2. Materials and methods

### 2.1. Experimental procedures

Details of the experimental procedures are provided in Chung (2011). In brief, seven participants with macular disease practiced reading for six training sessions. Participants S1–S6 were from the study of Chung (2011), while data from S7 has not been reported previously. Visual characteristics of these seven participants are given in Table 1. Before and after training, participants were tested on a battery of tests that included the measurements of visual acuity, the location of the preferred retinal locus for fixation, fixation stability, the critical print size for reading and the maximum reading speed (when print size is not a limiting factor). The post-pre changes of these tests, if any, were reported previously in Chung (2011). In this paper, we focus on reporting the changes of the psychometric functions as a result of perceptual learning.

**Table 1:** **Participant demographics**.

**Participant**	**Sex**	**Age**	**Diagnosis**	**LogMAR Acuity (RE, LE)**	**In 2011 study?**
S1	F	82	AMD	0.50 0.52	Yes
S2	M	85	AMD	0.70 0.74	Yes
S3	M	84	AMD	0.56 0.70	Yes
S4	F	73	AMD	0.66 0.48	Yes
S5	F	62	Stargardt	0.58 0.58	Yes
S6	M	57	Stargardt	1.10 1.10	Yes
S7	M	72	AMD	0.78 0.78	No

*All characteristics listed refer to the values during participation in the study*.

Reading performance was assessed using oral reading speed for single sentences presented in the RSVP format (Chung et al., 1998; Chung, 2002, 2011). On each trial, a single sentence was chosen randomly from a pool of 2630 sentences, containing between 8 and 14 words (mean = 10.9 ± 1.7 [SD]). All the words used were among the 5000 most frequently used words in written English, according to word-frequency tables derived from the British National Corpus (Kilgarriff, 1997). Words were rendered in Times-Roman font and were presented left-justified on a computer display, one word at a time in rapid succession, each for a fixed exposure duration. Participants were asked to read the words as quickly and as accurately as possible. The number of words read correctly was recorded after each trial. Feedback as to the number of words read correctly or the correct words (if read incorrectly) was not provided. In each block of trials, we used the method of constant stimuli to present sentences at five word exposure durations. The durations were chosen such that participants' reading accuracy spanned a range from 0–10% to 90–100% correct. Six sentences were tested at each duration, with a total of 30 sentences tested in each block and in a random order. With the exception of S6, all participants completed 10 blocks of trials (30 trials, or an average of ~330 words presented per block) in each of the six training sessions, for a total of 60 blocks. S6 completed only seven blocks in the first training session, and eight in each of the subsequent sessions, for a total of 47 blocks. Training sessions were scheduled once a week for six consecutive weeks for participants S1–S5. Due to unexpected illness and personal issue, there was a three-week gap between sessions 3 and 4 for S6, the rest of his training sessions also occurred on a weekly basis. S7's training occurred on a daily basis (due to availability of the participant). Previously we have reported that the improvements due to perceptual learning are similar whether training took place on a daily or a weekly basis (Chung and Truong, 2013). With the exception of the frequency of training sessions (daily vs. weekly), the training protocol was identical for all participants.

## 3. Results

### 3.1. Statistical modeling

To perform the statistical analyses subsequently described, we used the free software R (R Core Team, 2014). Additional analysis and plotting routines were written in Python, using the IPython (Pérez and Granger, 2007) environment and the NumPy/SciPy mathematics libraries (Millman and Aivazis, 2011).

There are several ways to analyze how the parameters of the psychometric function change over the course of training. The traditional approach (widely used, including in our own previous study), is to fit a psychometric function in each block as the first step, and then compare (i.e., with ANOVA, *t*-tests, etc.) or otherwise process the results of the fits (such as smoothing, fit an exponential, etc.) This two-step procedure is called the *Parameter-As-Outcome Model* (PAOM) in a recent article (Moscatelli et al., 2012) which serves as a tutorial to an alternative method comprising a principled one-step approach.

Using the one-step technique, psychometric functions are *simultaneously* fit to data and processed over time in a single step, permitting more robust statistical analyses. Since the change in performance over time is best described by an exponential function (Dosher and Lu, 2007; Chung, 2011), a non-linear mapping of the parameters vs. training block must be employed. No hypotheses exist about the change in slope over training, so non-parametric, assumption-free methods must be used. There are several possibilities, such as “additive models” (Wood, 2006), which have advantages over other non-parametric approaches such as LOESS or kernel regression (Knoblauch and Maloney, 2012). In a similar vein, we employed orthogonal polynomial fitting, where the change over time is modeled using sums of polynomials of increasing powers of the predictor variable (block number). Using R, this method can be incorporated as described above with simultaneous fitting of the psychometric function at each block, with the typical assumptions of a cumulative Gaussian psychometric function and binomial variance, such as in traditional probit analysis.

Two variants of this model were tested. The more general model (denoted M_*var*_) models both the slope and 50% point as arbitrary functions of the block number. An alternative model (M_*fixed*_), lets only the 50% point vary with the block; the slope is fixed. The sole free parameter when using orthogonal polynomials is the highest order of polynomial to utilize in fitting. Higher orders always yield a better fit to the data, but the risk is overfitting noise. To account for this, when choosing an order, a statistic such as BIC (Bayesian Information Criteria) is computed that penalizes the model likelihood by the number of parameters (Knoblauch and Maloney, 2012), here the highest order of polynomial. We summed the BIC across participants and evaluated all possible orders (1–60) of model M_*var*_. The minimum BIC occurred at order 2, meaning the slope and 50% point are best approximated by the sum of a linear term and a quadratic term. A third model (M_*exp*_) extended the traditional analysis (such as Dosher and Lu, 2007; Chung, 2011), modeling the change in performance as an exponential that reaches an asymptote, combined with a potential change in slope modeled as an arbitrary function, again using orthogonal polynomials.

### 3.2. Parameter estimates: changes in slope and 50% point

**Figure 2:**
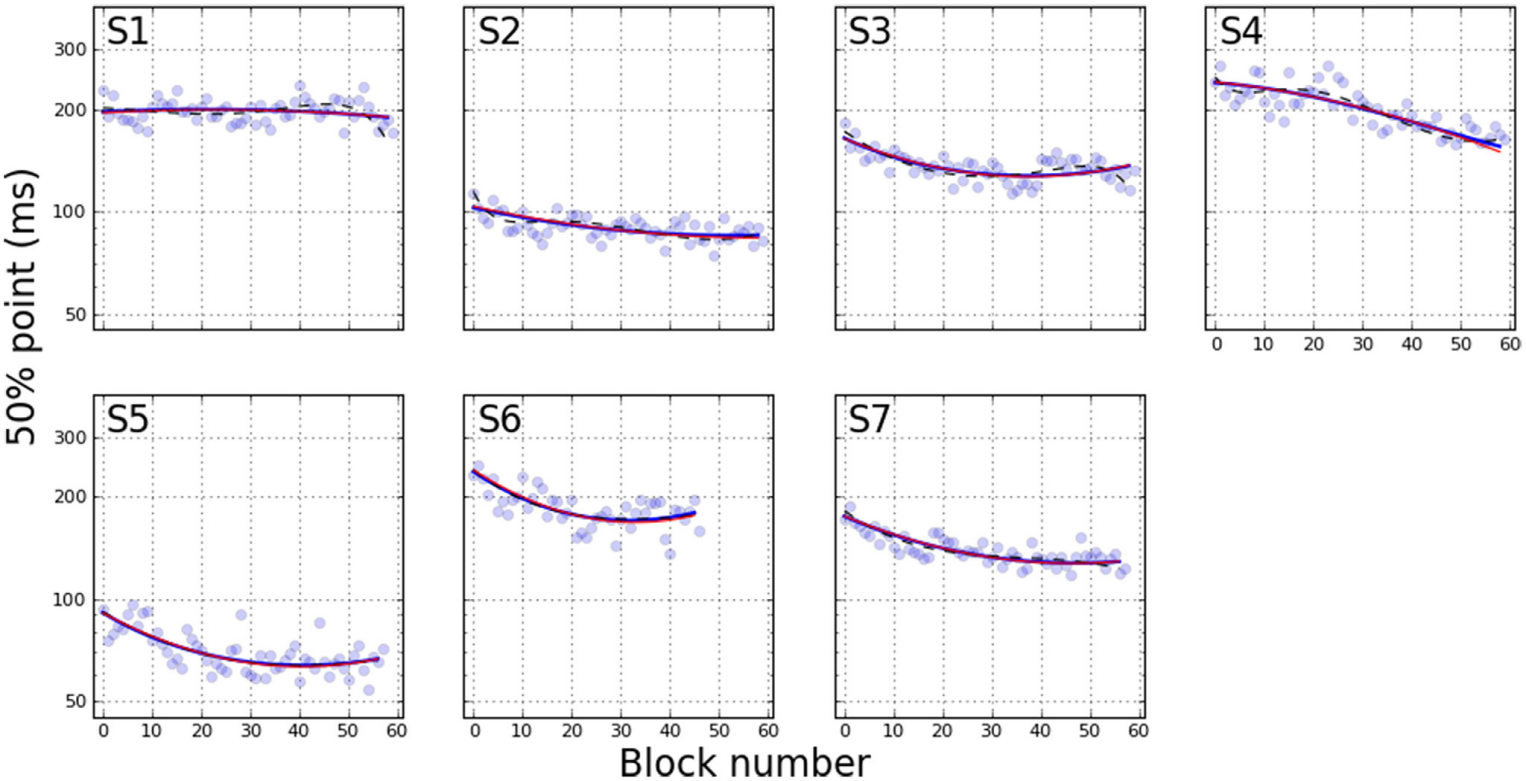
**50% point on psychometric function as a function of training block**. Blue curves are estimated using the variable slope model, while red curves indicate fixed slope. These curves are nearly indistinguishable. Dashed black lines show exponential fit. Pale blue dots represent PFs fit independently to each individual block.

**Figure 3:**
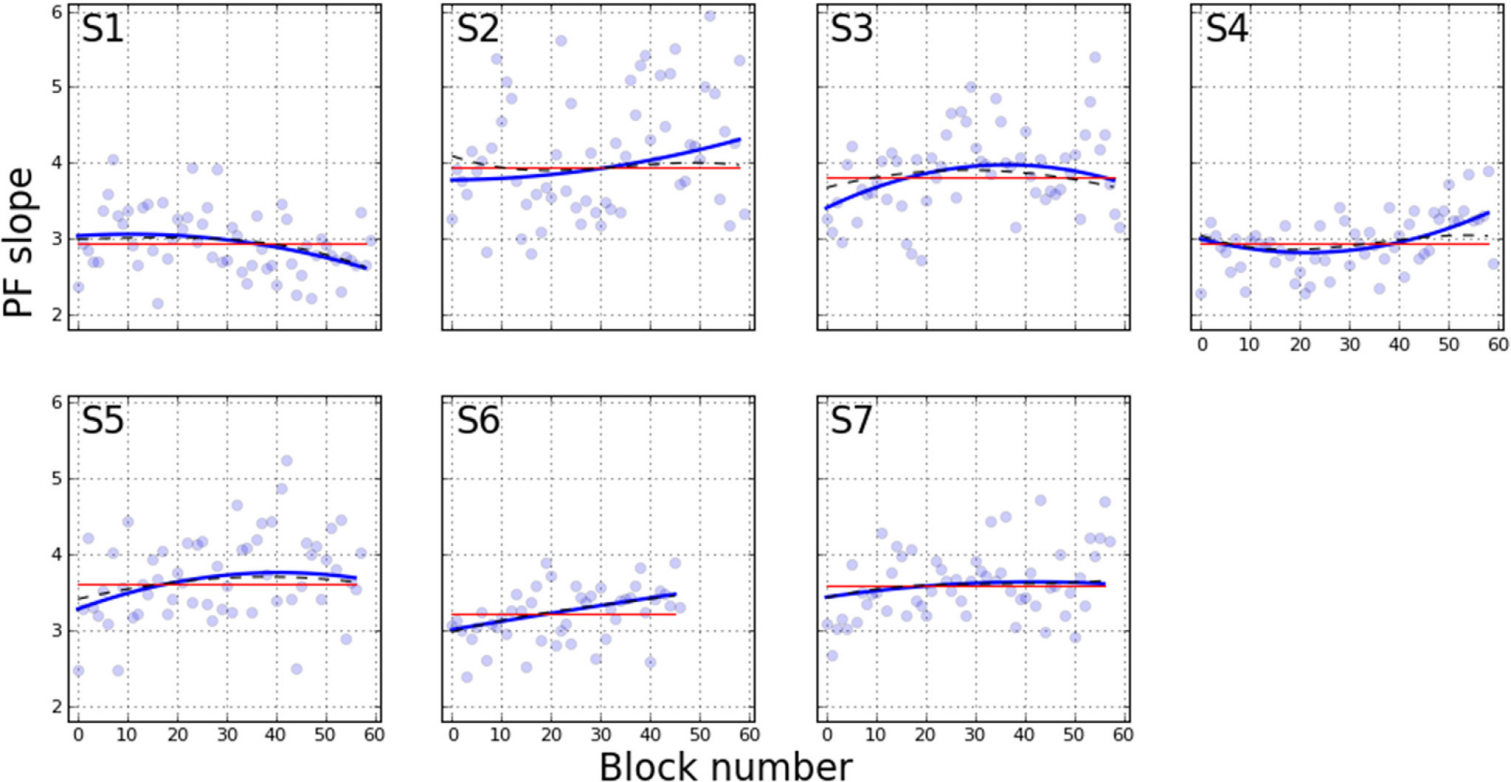
**Slope of PF across training blocks**. Curves are colored as in Figure 2, with the estimated slope parameter as the variable of interest.

Figures 2, 3 depict the estimated values for the 50% point and slope, respectively. Each line shows the fit based on the method indicated in the figure legend. Several qualitative observations can be made about Figure 2. First, despite the difference in the constraints of each of the three models, the 50% point estimates are remarkably consistent between models. Most participants show a decrease in stimulus duration corresponding to the 50% point over the duration of training. The decrease is generally asymptotic, resulting in a function that is concave upward. S1 shows little improvement, however, and S4 may not have reached asymptotic performance. Figure 3 demonstrates a general increase (steepening) in the slope of the psychometric functions, either asymptotic (S3, S5, S7), or increasing (S2, S4, S6). S1 is the only participant showing a decrease (shallowing) of the slope. These trends are generally consistent between the two models that let the slope vary, while the slope for model M_*fixed*_ is flat, by definition.

To compare with Chung (2011), Figure 4 plots the “reading speed” that is calculated from the estimated PFs. Reading speed is defined as the duration yielding 80% correct, converted to words-per-minute (wpm) by dividing the duration into 60 × 1000. This graph can be compared directly to Figure 1 of Chung (2011). For the present study, the only real divergence between the three models can be observed with S4. Here the orthogonal polynomials estimate the change in reading speed as an upward concave function, whereas the exponential fit models the change as a shallow linear curve. Another difference between the exponential fit and the other smoothing approaches is that S1 does not show an improvement in reading speed except for a steep rise in the first few blocks, which is best captured by the exponential.

**Figure 4:**
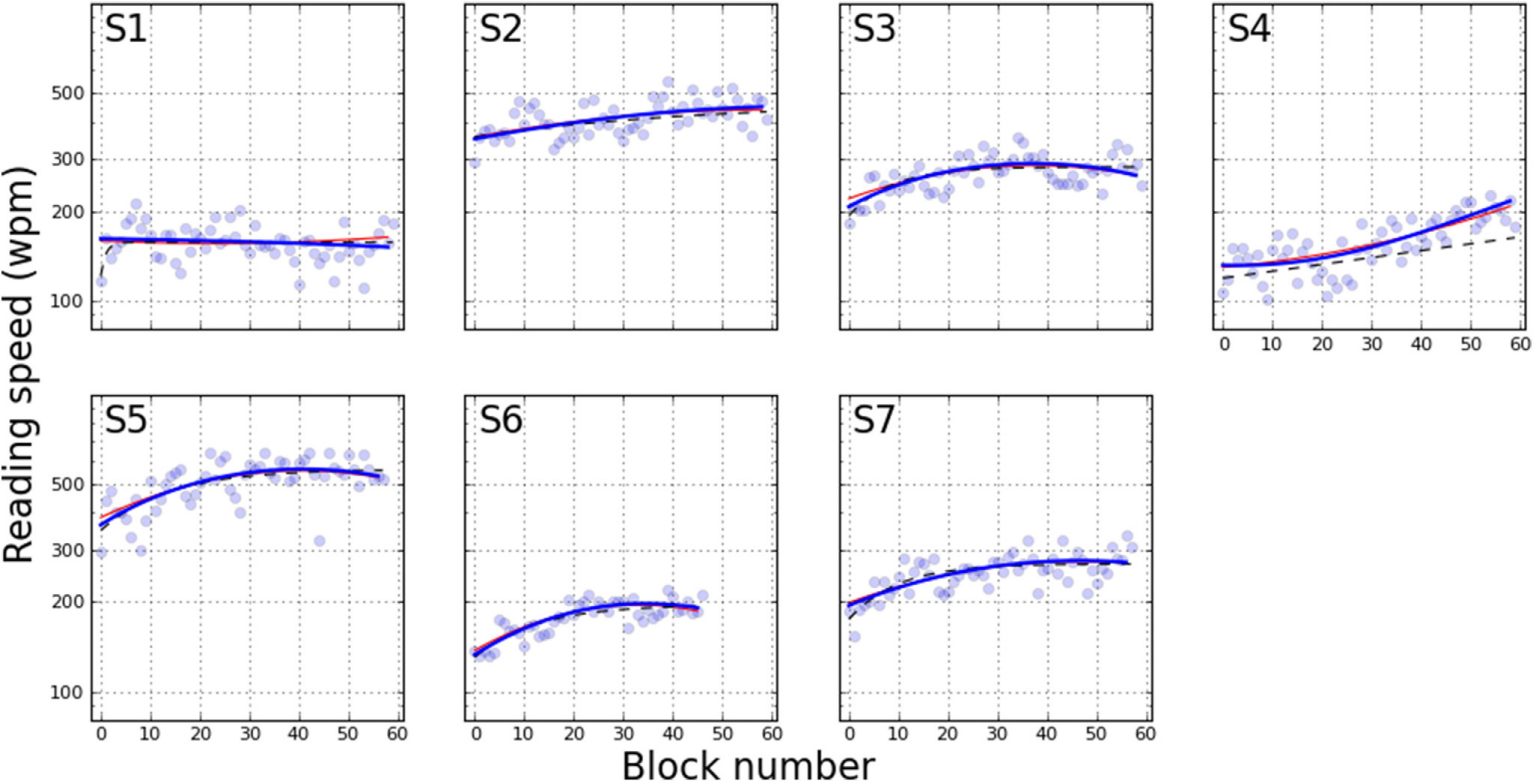
**Reading speed across training blocks**. The data is derived from Figures 2, 3, with conversion specified as reading speed $=\frac{60\times 1000}{80\%\text{accuracy level}}$.

The improvements due to perceptual learning can be quantified by comparing the fitted values from the first and last blocks, as shown in Tables 2–4. These tables demonstrate the similarity of the predictions of the three models, as well as indicating the ratio of improvement from first to last block. Table 4 further demonstrates the reasonable agreement in reading speed measurement with Chung (2011), despite the difference in methods.

**Table 2:** **Estimated first and last PF 50% points**.

**Participant**	**First block**			**Last block**			**Ratio $\left(\frac{First}{Last}\right)$**		
	^**M**^**var**	^**M**^**fixed**	^**M**^**exp**	^**M**^**var**	^**M**^**fixed**	^**M**^**exp**	^**M**^**var**	^**M**^**fixed**	^**M**^**exp**
S1	196.23	194.94	201.56	188.94	189.57	160.79	1.04	1.03	1.25
S2	102.58	103.40	113.95	85.16	83.58	85.85	1.20	1.24	1.33
S3	164.16	163.61	171.99	136.07	136.64	117.09	1.21	1.20	1.47
S4	239.02	239.86	247.82	155.30	149.64	162.96	1.54	1.60	1.52
S5	91.31	91.14	91.83	66.76	66.75	65.72	1.37	1.37	1.40
S6	237.02	239.22	239.57	179.43	176.35	176.26	1.32	1.36	1.36
S7	175.30	175.51	182.05	128.99	129.13	123.17	1.36	1.36	1.48

**Table 3:** **Estimated first and last PF slopes**.

**Participant**	**First block**			**Last block**			**Ratio $\left(\frac{First}{Last}\right)$**		
	^**M**^**var**	^**M**^**fixed**	^**M**^**exp**	^**M**^**var**	^**M**^**fixed**	^**M**^**exp**	^**M**^**var**	^**M**^**fixed**	^**M**^**exp**
S1	3.04	2.93	3.00	2.62	2.93	2.63	0.86	1.00	0.88
S2	3.77	3.94	4.09	4.31	3.94	3.97	1.14	1.00	0.97
S3	3.40	3.81	3.67	3.77	3.81	3.68	1.11	1.00	1.00
S4	3.00	2.94	3.04	3.34	2.94	3.04	1.11	1.00	1.00
S5	3.28	3.61	3.41	3.69	3.61	3.63	1.13	1.00	1.07
S6	3.01	3.22	2.97	3.47	3.22	3.45	1.16	1.00	1.16
S7	3.43	3.58	3.43	3.61	3.58	3.65	1.05	1.00	1.07

**Table 4:** **Estimated first and last reading speeds, calculated as estimated 80% point on PF converted to WPM using WPM $=\frac{60\times 1000}{duration}$. Columns marked (2011) show results of Chung (2011) study, which used a different method of fitting**.

**Participant**	**First block**				**Last block**				**Ratio $\left(\frac{First}{Last}\right)$**			
	^**M**^**var**	^**M**^**fixed**	^**M**^**exp**	**(2011)**	^**M**^**var**	^**M**^**fixed**	^**M**^**exp**	**(2011)**	^**M**^**var**	^**M**^**fixed**	^**M**^**exp**	**(2011)**
S1	161.55	158.96	155.91	118.52	151.38	163.46	178.50	172.81	0.94	1.03	1.14	1.46
S2	350.03	354.81	327.89	341.92	449.22	438.94	428.87	458.19	1.28	1.24	1.31	1.34
S3	206.87	220.43	205.83	189.08	263.77	263.94	302.67	285.91	1.28	1.20	1.47	1.51
S4	131.49	129.49	128.07	126.88	216.24	207.58	194.48	195.17	1.64	1.60	1.52	1.54
S5	363.64	385.03	369.99	349.78	531.45	525.69	535.64	595.51	1.46	1.37	1.45	1.70
S6	132.85	137.50	130.50	125.13	191.36	186.53	194.20	201.94	1.44	1.36	1.49	1.61
S7	194.64	198.87	187.18	n/a	271.99	270.30	286.43	n/a	1.40	1.36	1.53	n/a

**Figure 5:**
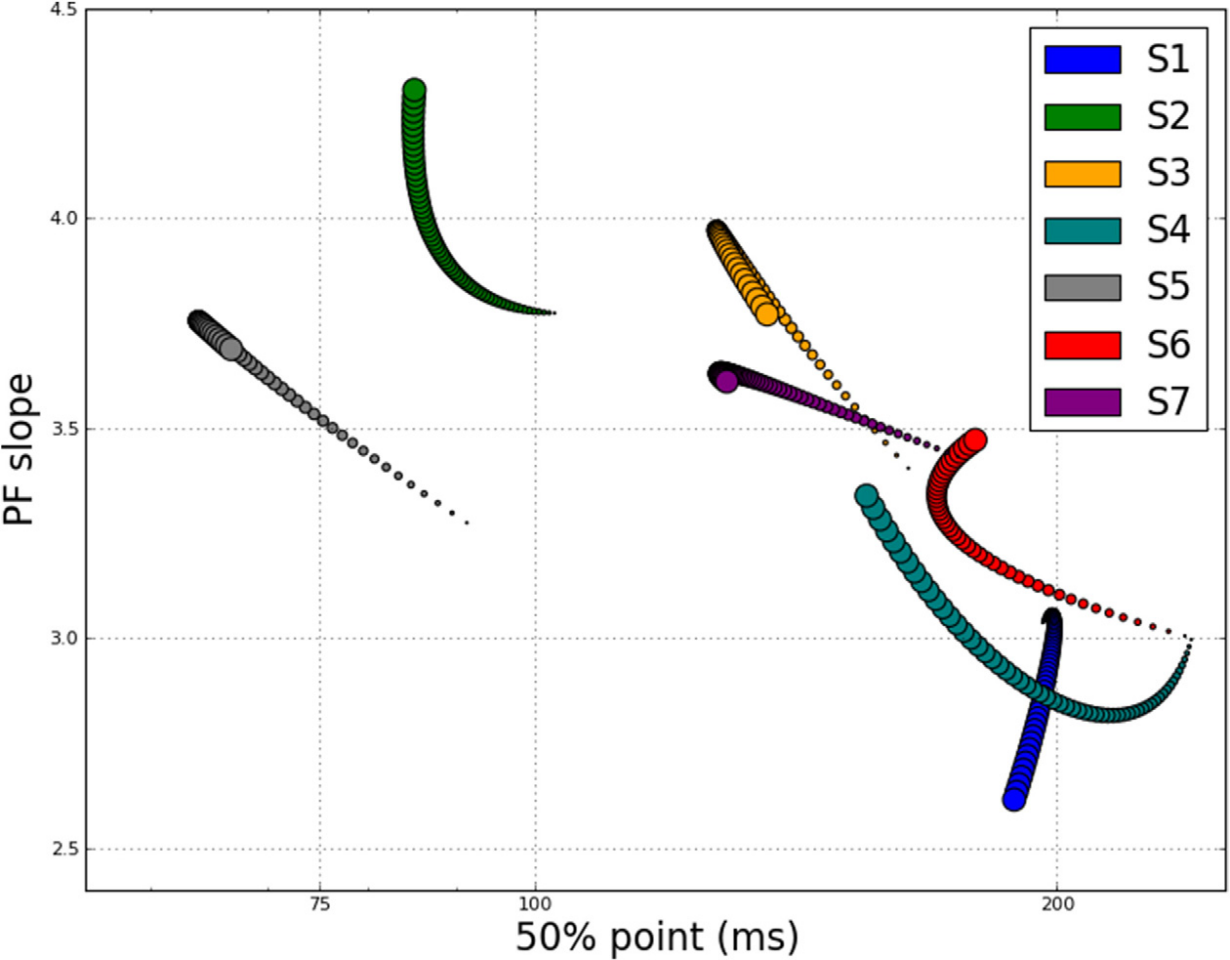
**Slope of PF vs. 50% point for each block of each participant, showing evolution throughout training**. Color indicates participant, with circle size going from small to large over course of training.

Until now, we have reported the changes in 50% point and the slope of the psychometric functions as separate entities, but since both parameters progressively change with training, are these values related? Figure 5 shows the corresponding changes to both the 50% point and psychometric function slope for all 7 participants on a single plot, as estimated using M_*var*_. Each marker indicates the value of the two PF parameters on one training block, with color indicating participant and symbol size going from small to large to indicate the progression of training blocks. For most participants, it is clear that a steepening of the psychometric function accompanied the observed decrease in 50% point, though there are significant individual differences.

### 3.3. Estimated change in performance across the PF

Changes in RSVP reading speed with training has been reported previously, but to our knowledge, whether or not the slope of the psychometric function changes with training has not been established. To confirm that the slope change is indeed significant, we performed statistical model comparison of the M_*var*_ and M_*fixed*_ models. Since M_*fixed*_ is a nested model of M_*var*_, a straightforward χ^2^ difference test can be used. With this analysis, the addition of the two slope terms (the linear and quadratic coefficients), was statistically significant for all participants except S7. (For this participant, note the flatness of the estimated slope in Figure 3). Table 5 lists the results of this test for each participant.

**Table 5:** **χ^2^ difference test of model *M_fixed_* vs. model *M_var_* for each participant. The nested model (*M_fixed_*) has two fewer degrees of freedom: linear and quadratic coefficients for the modulation of slope over the course of training**.

**Participant**	**ΔDeviance**	***p*-value**	**Significance**
S1	12.034	0.002	**
S2	7.997	0.018	*
S3	14.002	0.001	***
S4	11.777	0.003	**
S5	10.879	0.004	**
S6	7.617	0.022	*
S7	2.226	0.329	

The change in slope is consistent amongst observers except for S1 (opposite sign of slope change) and S7 (not statistically significant). It is well known that there is substantial individual variability in the effects of perceptual learning (Fahle and Henke-Fahle, 1996), therefore we are not surprised that not all participants showed the same effects. In fact, the percentage of our participants not showing the effect as the other participants (~28%) is comparable to the values reported for the percentage of participants not showing any improvement following perceptual learning (Fahle and Henke-Fahle, 1996; Chung et al., 2005).

**Figure 6:**
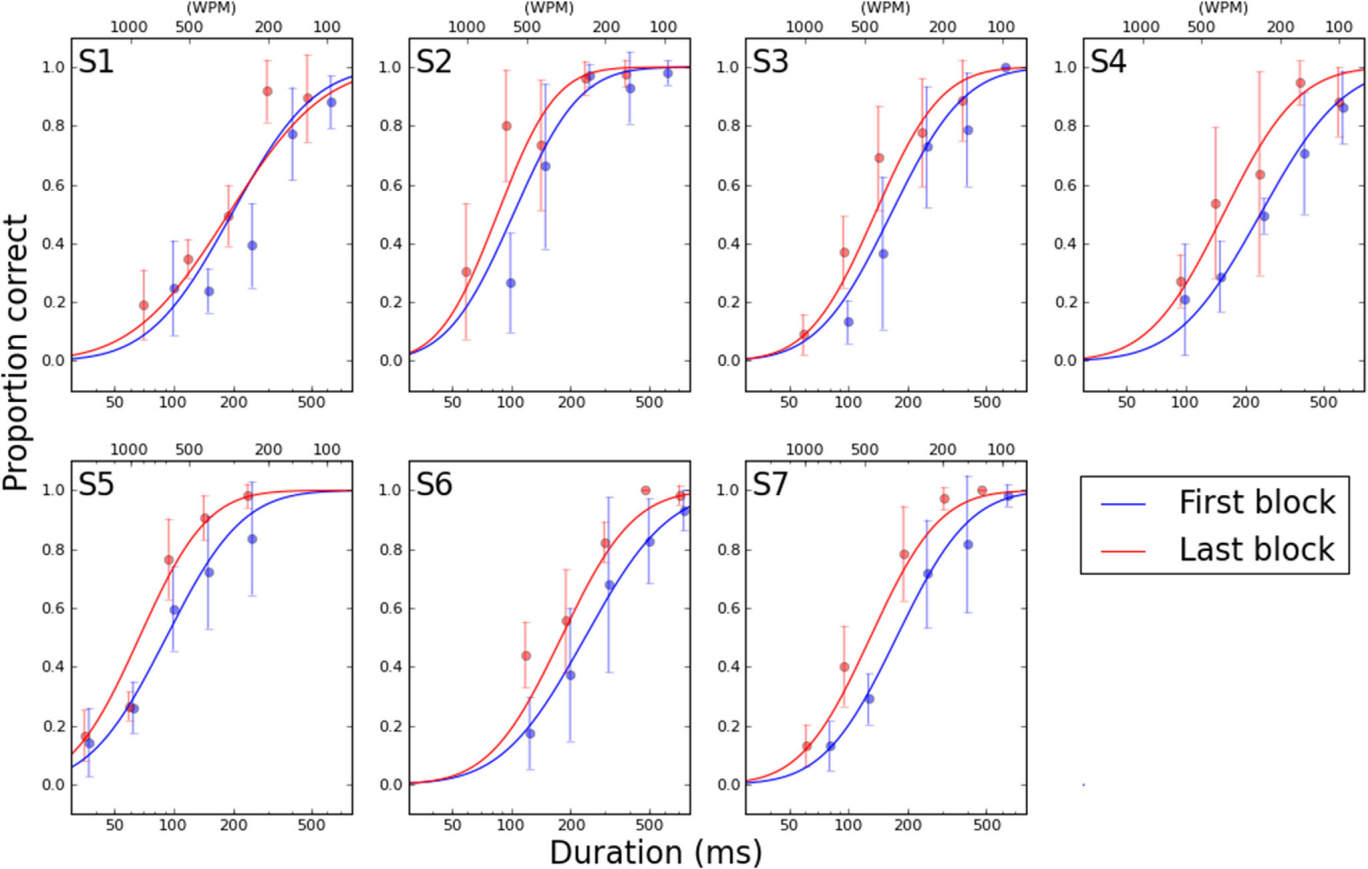
**First (blue curve) and last (red curve) psychometric functions for each participant, interpolated from model M_*var*_**. Abscissa is word duration, shown in milliseconds on the bottom axis and equivalent reading speed in words per minute on the top axis. Points show the mean and standard deviation of the six repetitions of each word duration in the specified block.

**Figure 7:**
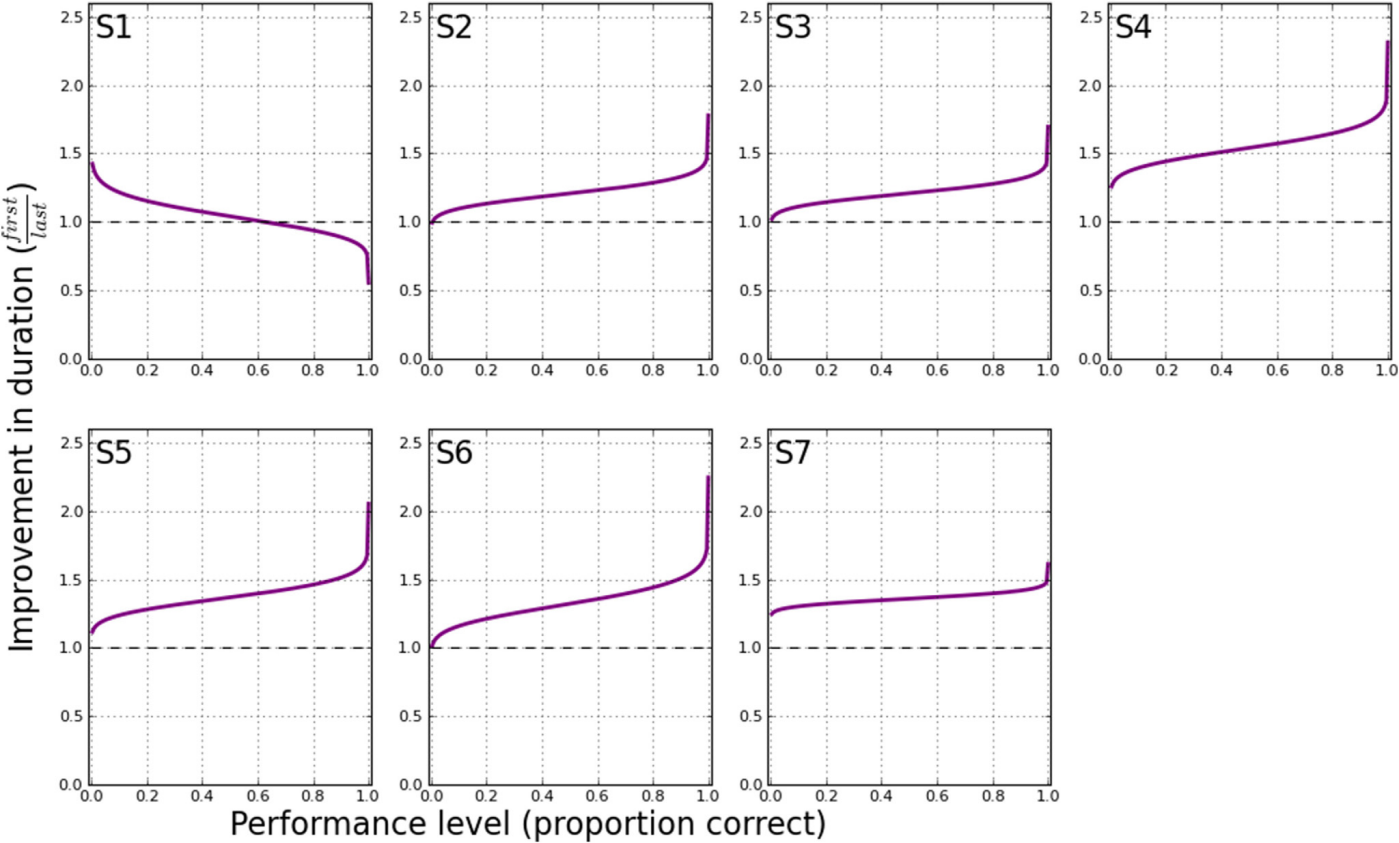
**Schematic improvement in performance (duration) at all points on the psychometric functions shown in Figure 6**. At a given performance level (proportion correct), the corresponding stimulus durations are determined that yield the specified performance. The ratio of these durations (first/last) is then plotted on the ordinate, at the performance level indicated by the abscissa.

To clearly illustrate the change in performance across the psychometric function, Figure 6 shows the first and last PF for each participant. The full PFs are shown (estimated using model M_*var*_), as well as the empirical data for the two blocks. From the PFs, the expected ratio of performance improvements can be estimated for any arbitrary criteria, as shown in Figure 7. Clearly, the more similar the slopes are, the flatter the ratio curve at different points of the psychometric function. In the case where the slope does not change (Figure 1A), the improvement curve will be completely flat (uniform improvements across PF), whereas for the steepening slope case (Figure 1C), the curve shown in Figure 7 will increase for higher performance levels (larger × values). Finally, the case of Figure 1B would result in a curve with lower performance levels worsening (ratio<1), and higher performance levels improving (ratio>1). For our participants, S2–S6 exhibited a pattern consistent with Figure 1C, in agreement with the slope ratios shown in Table 3. S1 showed a negative pattern, while S7's pattern was flat, more consistent with Figure 1A.

## 4. Discussion

In this paper, we performed detailed analyses of the rich data set of Chung (2011), with additional data from another participant with AMD, to address questions of whether there is a change in the slope of the psychometric function as a result of perceptual learning. With respect to this question, we had *a priori* reason to hypothesize that the slope of the psychometric function should either remain the same (but this should be accompanied by a shift of the psychometric function to reflect improvements in performance) or become steeper following perceptual learning. Here, the slope of a psychometric function represents the magnitude of the word duration that needs to be changed in order to alter the participant's reading accuracy by a certain amount. With perceptual learning, it is expected that participants would require a smaller change in word duration to produce the same amount of change in reading accuracy. With respect to our analysis, we found that the slope of the psychometric function became measurably steeper with training for 5 out of 7 participants (see Figures 4, 5), although we acknowledge that there are individual variabilities.

The change in the slope of the psychometric function is interesting, and may be able to account for some improvements in reading performance. However, if the psychometric function simply becomes steeper but does not exhibit a shift toward shorter durations, then the function would be pivoted at the 50% point (as shown in Figure 1B), and we should observe a *decrease* in reading speed for reading accuracy below 50%. Therefore, we also analyzed the data to determine if the improvements in reading performance occurred similarly across all durations, or only for some specific durations. As shown in Figure 7, the improvements in reading performance are not the same across all durations, ruling out scenario A in Figure 1 as the outcome of perceptual learning for most of the participants (S2–S6). The improvements are slightly larger near the high-end (performance close to 100% accuracy) of the psychometric function than the low-end (performance close to 0% accuracy). This finding, combined with the steepening of the psychometric function, identify scenario C as the effect of perceptual learning on the psychometric functions for our reading data.

In summary, following an RSVP training task to train participants with macular disease, we found that in addition to the previously reported improvement in reading speed, defined at the 80% accuracy, there is a steepening of the psychometric function relating reading accuracy with word exposure duration, accompanied by a shift of the psychometric function toward shorter duration. The shift is such that the psychometric function now appears to be pivoted at the low-end of the function. As such, the magnitude of improvement in reading speed would depend on the criterion to define reading speed. For example, the improvement is generally slightly greater when reading speed is defined at 80% accuracy than at 50%. This point is important for studies that use adaptive methods such as staircases for training, where reading performance is determined for more or less a similar accuracy level. Depending on the criterion accuracy level chosen, a larger or a smaller magnitude of improvement may be observed, and comparisons across studies would need to ensure that the accuracy levels are comparable.

### Conflict of interest statement

The authors declare that the research was conducted in the absence of any commercial or financial relationships that could be construed as a potential conflict of interest.
